# Characteristics and outcome of children with juvenile dermatomyositis in Cape Town: a cross-sectional study

**DOI:** 10.1186/s12969-016-0118-0

**Published:** 2016-11-11

**Authors:** Lawrence Owino Okong’o, Monika Esser, Jo Wilmshurst, Christiaan Scott

**Affiliations:** 1 Paediatric Rheumatology Service, Department of Paediatrics and Child Health, Red Cross War Memorial Children’s Hospital and the University of Cape Town, Cape Town, South Africa; 2 Department of Paediatrics and Child Health, University of Nairobi, Nairobi, Kenya; 3 NHLS, Tygerberg Hospital and Stellenbosch University, Stellenbosch, South Africa; 4 Paediatric Neurology Service, Department of Paediatrics and Child Health, Red Cross War Memorial Children’s Hospital and the University of Cape Town, Cape Town, South Africa

**Keywords:** Juvenile dermatomyositis, JDM, Outcome, Africa

## Abstract

**Background:**

Juvenile dermatomyositis (JDM) is a rare idiopathic inflammatory childhood myopathy of uncertain aetiology. The demographic and clinical presentation of JDM may differ by race and geographic regions. Few studies have described the characteristics of JDM patients from Africa.

**Methods:**

We conducted a retrospective observational study to determine clinical characteristics and outcomes of patients satisfying the Bohan and Peter criteria for probable JDM seen between 2004 and 2013 in three hospitals in Cape Town, South Africa.

**Results:**

Twenty five cases were identified: 16 female and 9 male; thirteen (52 %) were of indigenous African, eleven (44 %) mixed and one (4 %) European ancestry. The median ages at disease onset and diagnosis were 6.75 (range 2.0–9.7) and 7.9 (range 3.4–9.75) years respectively. Eleven patients had calcinosis while the mortality was 2/25 (8 %). Only 40 % of the patients had clinically inactive disease by PRINTO criteria (modified) at last review. There was no statistically significant difference in racial distribution (*p*-value = 1), age at disease onset (*p*-value = 0.87) and disease duration prior to treatment initiation (*p*-value = 0.75) between patients who had clinically active and inactive disease.

**Conclusion:**

The demographic characteristics of children with JDM were similar to that from most other regions of the world with female predominance and similar age at onset. Majority of the patients remained with clinically active disease, which put them at risk of further disease complications. Long term follow up and use of appropriate treatment guidelines may be indicated in management of JDM patients for optimum treatment outcomes.

## Background

Juvenile dermatomyositis (JDM) is an immune mediated vasculopathic disease of childhood characterized by inflammation of the striated muscles, skin and internal organs. The most common clinical manifestations are proximal muscle weakness and characteristic cutaneous lesions such as heliotrope rash, Gottron’s papules and calcinosis. However, disease manifestations may occur in the lungs, heart, gastrointestinal tract and other organs. The pathogenesis of JDM is not fully understood but environmental factors are thought to trigger the disease in genetically predisposed children [1–4].

JDM is most commonly diagnosed and classified using the Bohan and Peter criteria published in 1975 [5, 6]. The criteria include presence of characteristic rashes with any of symmetrical proximal muscle weakness, elevated serum muscle enzymes, electromyographic changes and features of inflammatory myositis on muscle biopsy. The presence of the skin manifestations together with two of the other features is required for classification of a patient as having probable JDM. The characteristic skin lesions may also occur without apparent muscle involvement and is referred to as amyopathic dermatomyositis [7].

Data from multicenter patient registries indicate that the average age of onset of JDM is seven years with greater incidence in girls than boys (ratio of 2:1) [8–11]. However, regional differences exist and studies from India and Saudi Arabia have reported higher incidence rates in male children [12, 13]. JDM occurs in all regions of the world though some studies suggest that there could be differences in incidence rates among different racial groups. Mendez et al. reported a lower incidence rate among children of Hispanic ancestry compared to those of African and Caucasian ancestry in the USA [14]. Racial differences have also been noted in the incidence rates of certain disease manifestations with much higher incidence of calcinosis being reported among children of African ancestry compared to other racial groups [9, 15, 16].

The treatment outcome of JDM has improved over the years with the mortality rate falling significantly from more than 30 % before routine use of steroids [17], to less than 5 % in most recent studies [11, 18–20]. Despite the improvement in mortality rates among JDM patients, disease and treatment related damage remains a major challenge and significantly affect the quality of life of affected individuals. However, data on the characteristics and treatment outcome of JDM patients from Africa is scarce and only one study has been published that specifically described a series of JDM patients from the continent [9]. In that study, a higher rate of calcinosis and vasculitis compared to that from other regions was reported suggesting that the clinical manifestations and outcome of JDM in African patients could be different.

We therefore carried out a study to review and describe the clinical features and treatment outcomes of patients seen with JDM from Cape Town, South Africa.

## Methods

### Study design, study setting and patients

We carried out a retrospective folder review of JDM patients seen between January 2004 and December 2013 in three tertiary care hospitals (Tygerberg, Groote Schuur and Red Cross War Memorial Children’s Hospital) in Cape Town, South Africa. These three hospitals are the main tertiary referral hospitals for the Western Cape Province and also serve the surrounding regions of the Eastern and Northern Cape provinces of South Africa. Patients from this catchment area diagnosed with JDM were likely to have been referred to one of these centres for evaluation and treatment. The approximate population in the primary catchment area was 4.6 Million in 2004 and 6.1 million in mid-2014 with children (people aged <15 years) constituting 35 and 26.5 % of the population respectively [21]. The province populace is composed of people of diverse ancestral backgrounds with the most populous groups being, in descending order, people of mixed, indigenous African and European ancestries [21].

Only patients who were seen between January 2004 and December 2013 and satisfied the Bohan and Peter criteria for classification as probable or definite JDM were included. Data was extracted from the patient records as at the last review prior to 31^st^ December 2014 to allow for a minimum follow up period of one year for each patient. Data was abstracted onto case record forms (CRF) and later transcribed onto an excel spreadsheet. Information collected included: demographics, clinical and laboratory features, radiological investigation results, treatment given and treatment outcomes.

The primary outcome variable was disease activity at last review. Patients were classified as having clinically active disease or inactive disease. A modification of the PRINTO criteria for inactive disease [22] was used. The PRINTO criteria include creatine kinase (CK) < 150 U/L, childhood myositis assessment scale (CMAS) > 48, Manual muscle testing score (MMT) > 78 and Physician global (VAS) <0.2. Patients satisfying any three of these criteria are classified as having clinically inactive disease. We used a modification of these criteria due to difficulties with direct comparisons since this was a retrospective study and data on some of the PRINTO criteria items was not available. Therefore for this study we modified the criteria as follows (modification in brackets): CK < 150 U/L (No modification), CMAS > 48 (No modification), MMT > 78 (or documented full muscle strength using any muscle strength assessment scale by the attending physician) and Physician global (VAS) <0.2 (or documentation of absence of symptoms and signs of active disease by the clinician).

Patients were classified as having inactive disease if they satisfied any three of these criteria. Secondary outcome measure was damage defined as persistent changes in anatomy, physiology or function present for at least 6 months at the last review (IMACS definitions of damage and specific damage items) [23, 24]. Damage was assessed as being present or absent by systems and patients classified as either exhibiting damage (any) or none at last follow up.

### Data analysis

Data was analysed using R i386 3.1.0 (The R Foundation for Statistical Computing, Vienna, Austria) software. Median and interquartile ranges were used for descriptive statistics for quantitative variables (non-normally distributed data). Frequencies were computed for qualitative variables. Non-parametric tests (Wilcoxon rank sum tests) were used for comparison of medians across groups. For comparison between categorical variables, cross-tabulation with formulation of Fisher’s exact (chi square) statistic was used. The two sided *p*-values <0.05 were considered to be statistically significant.

The study was approved by the human research ethics committees of Cape Town (HREC/REF: 062/2014) and Stellenbosch universities (S14/04/080).

## Results

### Patient characteristics

Twenty seven cases with a diagnosis of JDM seen between 2004 and 2013 were identified. Two had amyopathic JDM and were excluded as they did not meet the inclusion criteria of probable JDM. Of the other twenty five, twelve satisfied the criteria for classification as definite JDM. Six of the thirteen patients with probable JDM also had features of muscle inflammation on magnetic resonance imaging (MRI). Sixteen of the patients were female (F:M 1.8:1). Thirteen (52 %) of the cases were of indigenous African, eleven (44 %) mixed and one (4 %) European ancestry.

The median age at disease onset was 6.75 (range 2.0–9.7) years and 7.9 (range 3.4–9.75) years at diagnosis. The median duration from symptom onset to diagnosis was 4 (range 0.5–84) months. One patient with gottrons papules, calcinosis, elevated muscle enzymes, severe proximal weakness and arthritis was retrospectively diagnosed with JDM by the paediatric rheumatology team after a period of 84 months having previously been labelled as discoid lupus.

### Diagnostic features and characteristics

**Fig. 1:**
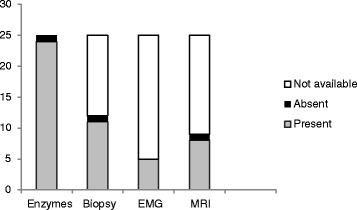
Diagnostic investigation results. Present: features supportive of myositis; Absent: not supportive of myositis; Not available: Not done or results not accessible. Muscle enzymes were elevated in 24 of the 25 patients

Muscle weakness and characteristic cutaneous manifestations were documented in all the 25 patients. Heliotrope rash occurred in 20 and Gottron’s papules in 19 of the patients. Muscle enzymes were elevated in all but one patient. This patient however, had suggestive muscle biopsy changes and satisfied the criteria for classification as probable JDM. Creatine kinase (CK) was elevated in 22 of 24 cases. The two patients with normal CK had elevated AST and ALT and in addition one had elevated aldolase and the other had elevated lactose dehydrogenase (LDH). Aldolase was only determined in five patients and was elevated in three of them. Figure 1 summarizes results of the diagnostic procedures and criteria.

Muscle biopsy was performed in twelve (48 %) of the patients and showed characteristic features including perifascicular atrophy, fibre degeneration and regeneration and perivascular inflammation consistent with JDM in 11 (91.7 %) of the cases.

Five of the patients had documented EMG results showing features consistent with myositis. Muscle MRI for initial diagnostic work-up was done in nine patients and demonstrated active inflammation in eight of the patients. All the patients in whom MRI was done were diagnosed in the latter half of the period under review (2009–2013).

### Clinical characteristics

There was no documented family history of neuromuscular or autoimmune diseases among the cases. However, history of preceding infection was reported in 6 cases: upper respiratory tract infection (URTI) in 4 cases and dental or skin abscesses in 2 cases with a median time to onset of JDM symptoms of 1 month (range 1 week – 3 months). One patient had a traumatic event (sexual abuse) one month prior to onset of JDM symptoms.

Pulmonary involvement was reported in four patients and two of them died; one with suspected severe interstitial lung disease and the other with pulmonary hemorrhage and sepsis. The third patient had clinical as well as high resolution computerized tomography (HRCT) scan features of interstitial lung disease (ILD) and restrictive pattern on pulmonary function tests (PFTs). One other patient had deranged spirometry results, which normalized with treatment. Antisynthetase antibodies (anti Jo-1) were tested in two of these four patients and was negative in both of them. In total, PFT results were available for six patients (done after mean of 4.4 years from diagnosis) and only two of the patients had deranged lung function parameters (restrictive pattern).

Cardiovascular involvement was observed in one patient who presented with generalized oedema and heart failure. Echocardiography showed mildly dilated chambers with reduced ejection fractions (EF) of <55 %. This patient had clinical resolution of symptoms as well as improvement on the EF on echocardiography. Echocardiography however, was not routinely done to document baseline state or monitor cardiac function of JDM patients in the three centres. Neurological manifestations were seen in two cases; one had convulsions thought to be due to cerebral vasculitis and the other had localized sensory neuropathy.

**Fig. 2:**
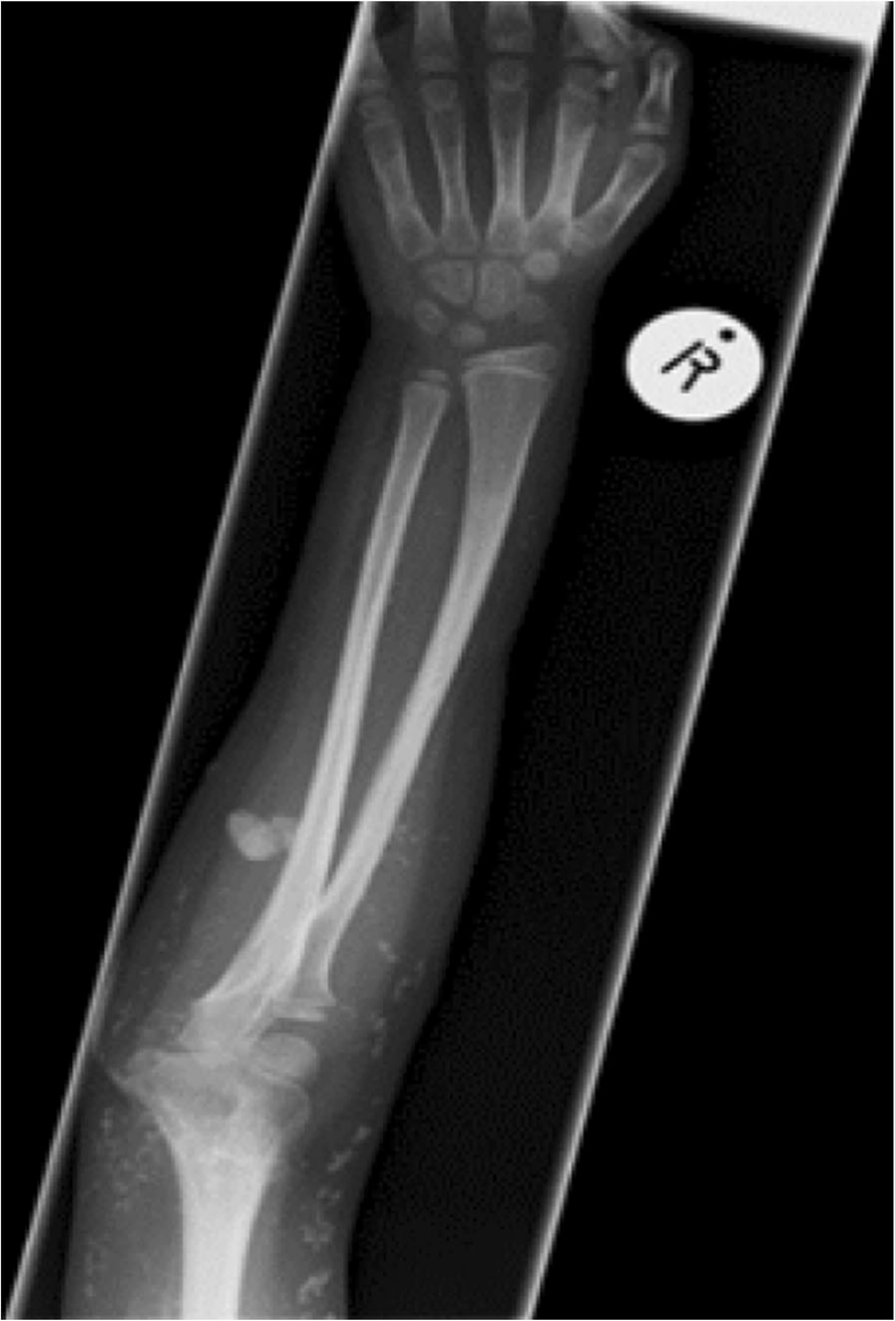
Forearm radiograph showing mixed tumoural and planar calcinosis in one of the JDM patients

Cutaneous and musculoskeletal manifestations were the most common and are summarized together with other clinical features in Table 1. Of note, three patients had calcinosis at diagnosis and eight others developed calcinosis during follow up. Calcinosis resolved in three patients during follow. Figure 2 shows a radiograph of one of the patients showing tumoural and planar calcinosis in the forearm. There was no statistically significant difference in the age at onset, age at diagnosis and duration of follow up among patients who developed calcinosis and those who did not have calcinosis. The median time to diagnosis from symptom onset was longer in children who had calcinosis (6 months) than in those who did not have calcinosis (3 months). The difference was however not statistically significant (*p* = 0.13). In addition, even though there was a tendency towards a higher prevalence of calcinosis among indigenous African children (8/13) compared to children of other racial backgrounds (3/12), the difference was not statistically significant (*p* = 0.11).

**Table 1:** Disease manifestations among the JDM cases in Cape Town

	Cumulative clinical manifestations (%)	Clinical manifestations at last review
Skin		
Calcinosis	11 (44)	8 (32)
Skin ulcers	11 (44)	1
Oedema (Generalized, Periorbital or limb)	11 (44)	0
Alopecia	3 (12)	3 (12)
Lipodystrophy	1 (4)	1 (4)
Musculoskeletal		7 (28)
Muscle tenderness	13 (52)	0
Arthritis	7 (28)	2 (8)
Contractures	5 (20)	5 (20)
Osteoporosis with fracture	1 (4)	1 (4)
Endocrine	9 (36)	9 (36)
Growth failure	8 (32)	8 (32)
Diabetes mellitus	1 (4)	1 (4)
Adrenal insufficiency	1 (4)	0
Respiratory	3 (12)	3 (12)
Interstitial lung disease (ILD)	2 (8)	2 (8)
Dysphonia	2 (8)	0
Pulmonary hemorrhage	1 (4)	1 (4)
Gastrointestinal		
Dysphagia	5 (20)	0
Abdominal pain or bleeding	3 (12)	0
Ocular	2 (8)	2 (8)
Cataracts	2 (8)	2 (8)
Cardiovascular		
Abnormal capillaroscopy	15 (60)	NA
Raynaud’s phenomenon	6 (24)	NA
Cardiomyopathy	1 (4)	0
Nervous system	3 (12)	1 (4)
Seizures; neuropathy	2 (8)	0
Sensorineural hearing loss	1 (4)	1 (4)
Infections		
Fungal (skin)	7 (28)	NA
Bacterial (staph aureus 2, gram negative 1)	3 (12)	NA
TB	2 (8)	NA

### Laboratory investigations

Among the MSAs, only anti-Jo1 was tested and was negative in all the six patients tested. ANA results were available in 19 patients and was low positive in seven (three had Hep-2 titres of 40, 40 and 100; and four had composite ANA ratio of 8, 2.2 4.6 and 32 U/L). The results of the laboratory investigations are summarized in Table 2.

**Table 2:** Laboratory investigation results

Investigation	Reference range	Median (IQR)	No. positive/No. tested	Percentage
Enzymes				
CK	26–145 U/L	1074.0 (222.8–3397.5)	22/24	91.7
Aldolase	3.0–12.0U/L	12.6 (7.7–15.9)	3/5	60
LDH	142–261U/L	445.0 (277.0–493.5)	14/19	73.7
AST	0–41 U/L	74.0 (35.25–140.5)	16/22	72.7
ALT	5–25 U/L	48.0 (26.5–82.0)	18/23	78.3
Autoantibody				
Anti-Jo1	<7 EliA U/ml	0.3 (0.3–0.45)	0/6	0
Anti-RNP	<7 EliA U/ml	0.3 (0.3–0.5)	0/9	0
Inflammatory markers				
ESR		20 (12–35)	11/19	57.9
CRP		2.6 (<1–6)	2/10	20
vWF activity		77 (62–112 %)	2/4	50

### Treatment

**Fig. 3:**
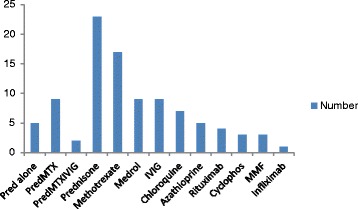
Drugs used in management of Cape Town JDM patients. Abbreviations: Cyclophos: cyclophosphamide; Medrol: intravenous methylprednisolone; Pred: Prednisone PredMTX: Prednisone and methotrexate; IVIG: Intravenous immunoglobulin

Information on treatment was available for 23 of the 25 patients. We could not locate treatment records in two cases. The most common drugs used were oral prednisone (100 %), methotrexate (74 %), intravenous methylprednisolone (39 %) and intravenous immunoglobulin (IVIG, 39 %). Prednisone alone was used in five patients, prednisone and methotrexate without another agent in nine patients and various other drugs were added as second line drugs to the main treatment modality (prednisone and methotrexate) in poor responders as summarized in Fig. 3 below. All the patients received corticosteroids during the course of their treatment. Biologics were used in 6 patients (rituximab in 5 and infliximab in 1). Oral steroids were given at a dose of 2 mg/kg, though there was no uniformity on when to start to taper the dose and over what duration of time. The patient who received infliximab had defaulted from follow up after one dose and was yet to return for evaluation and further doses by the time of completing data collection and analysis.

### Treatment outcome: diseases activity, mortality and damage

Of the 25 patients, 10 were still in care, 8 had been discharged, 2 transferred to other centres, 3 were lost to follow up and 2 died. Ten (40 %) of the patients had clinically inactive disease. There was no statistically significant difference in the median duration of follow up (*p* = 0.28), age of onset, race, diagnostic delay and disease category (probable or definite) between those who had clinically inactive and active disease. These findings are summarized in Table 3 below.

**Table 3:** Determinants of disease activity

	Clinically inactive disease	Clinically active disease	*p*-value
Number	10	15	
Sex (F/M)	8/2	8/7	*p* = 0.228^a^
Race (Indigenous African/other)	5/5	8/7	*P* = 1
Age at onset (Median) years	7.8	6.0	*p* = 0.868^a^
Time to diagnosis (Median) months	3.5	4.5	*p* = 0.745^a^
Duration follow up (Median) months	55.5	49.1	*p* = 0.28^a^
Calcinosis (Yes/No)	4/6	6/9	*p* = 1^b^

^a^Wilcoxon rank sum test, ^b^Fisher exact

The mortality rate was 2/25 (8 %) over a median follow-up period of 50 months. Death resulted from respiratory failure in the setting of global weakness (and possible interstitial lung disease) in one patient and from sepsis, GI bleed and pulmonary hemorrhage (evident at autopsy) in the other.

Overall, 18 (72 %) of the patients had some documented damage though a full damage assessment was hampered by missing data occasioned by the retrospective nature of the study. The most common systems in which disease damage was reported were: endocrine (9 cases), skin (8 cases), musculoskeletal (7 cases) and respiratory (3 cases) systems. Endocrine damage was mainly as a result of growth failure in 9 (40 %) of the patients though one patient developed treatment (steroid) associated diabetes mellitus and another, adrenal insufficiency. Growth failure was defined by the IMACS criteria as presence of two of the following three features: a. Less than 3 percentile height for age, growth velocity over 6 months less than 3 percentile for age and growth curve crossing at least 2 centiles (5 %, 10 %, 25 %, 50 %, 75 %, 95 %) on growth chart.

Musculoskeletal damage included one patient who had osteoporosis with vertebral fracture that was demonstrated using bone scans (scintigraphy). One patient, who had suffered from suppurative otitis media during follow up, developed sensorineural hearing loss. Three patients had calcinosis at diagnosis, 8 developed calcinosis and three had resolution of the calcinosis during follow up.

## Discussion

A better understanding of the epidemiology and clinical outcomes of JDM has been enhanced by the establishment of multicenter and multinational collaborations such as the paediatric rheumatology international trials organization (PRINTO) and international myositis assessment and clinical studies group (IMACS). However, data from Africa has been scarce and the epidemiology of JDM from the continent has been based on extrapolation of data collected from other populations and regions. This study adds to the available data on the epidemiology and treatment outcome of JDM in Africa.

The median age of onset (6.75 years) and gender composition (female: male 1.8:1) among the JDM patients in our study was similar to that reported by most studies from other regions with a mean age at disease onset of seven years and a female predominance of about 2:1 [10, 11, 20]. In the current study, we found a relatively high rate (44 %) of calcinosis. Children of indigenous African ancestry appeared to have a higher prevalence of calcinosis (61.5 %) compared to patients of other ancestral background (25 %), though the difference was not statistically significant (*p* = 0.11). Higher rates of dystrophic calcinosis have been reported among indigenous African compared to JDM patients of other ancestral backgrounds [16]. It is not clear why indigenous African patients appear to have higher risk of calcinosis.

Certain immune phenotypes associated with presence of anti-MJ autoantibodies, found in 12–23 % of JIIM patients, have been associated with higher risk of occurrence of calcinosis and severer disease [25]. It is not known if black African children have higher prevalence of these immune phenotypes thus putting them at greater risk for calcinosis. The immune phenotype was not determined for our patients and published studies are not available on the comparative prevalence of autoantibody and other associated risk factor profiles among indigenous African compared to children of other ancestral backgrounds.

Increased risk of calcinosis and other disease damage could also be related to poor access to care with subsequent inappropriate and delay in instituting treatment. Delay in diagnosis may be the consequence of lack of access to clinicians skilled in diagnosing and managing dermatomyositis and similar conditions; and lack of access to diagnostic facilities such as EMG and muscle biopsy. However, there was no significant difference in the delay in making a diagnosis between the patients of indigenous African and patients of other ancestral origins (*p* = 0.27).

As the search for better and more acceptable diagnostic procedures and tools continues, MRI is gradually being used in the three centres instead of or in addition to EMG and muscle biopsy for diagnosis of JDM. Nine muscle MRIs were done as part of the diagnostic evaluation of children; all in the latter half of the period under review. Thus MRI seems to have gained prominence as a diagnostic tool for JDM consistent with findings from other studies [19, 26, 27]. However, in Africa and many other resource constrained economies, MRI may not be available in many centres and even where it is available, long waiting times, technical issues with reporting and cost implications may prove prohibitive. Thus additional diagnostic modalities such as muscle ultrasound and Bone scintigraphy that may provide cheaper and more convenient alternatives for demonstrating evidence of muscle inflammation may be welcome [28–30].

The mortality rate (8 %, 1 death per 52 patient years) in our series appears higher than the 2–4 % reported in most recent studies from other regions [11, 18–20]. This finding was consistent with that by Faller et al. [9] who reported a mortality of 9.5 % (1 death per 27.3 patient years) among indigenous African children with JDM from Johannesburg, South Africa. These two studies were small and it’s not possible to draw generalizable conclusions. They however provide some insight into possible treatment outcomes of JDM in Africa. Two deaths were reported in our study as well as in the study by Faller et al. and all the four deaths resulted from respiratory (involvement) disease which has been identified as an early disease feature predictive of mortality [18].

Juvenile dermatomyositis is a chronic disease and many patients may never achieve a clinically inactive disease state. After a median follow up time of 50 months, only ten (40 %) of the 25 patients had clinically inactive disease (modified PRINTO criteria) at their last follow up. Sanner et al. studied the long-term outcome of JDM and after 16.8 years, 59 % of the patients still had clinically active disease [31]. Similarly, Sun eta al analyzed the treatment outcome among JDM patients over a ten year period in a Taiwanese hospital found that only 33 % among the cohort of 39 patients were symptom free at last review [32]. It is thus clear that most children with JDM have long-term inflammation and disease activity that linger long after initiation of treatment. Formulation of appropriate guidelines and standard of care protocols to guide short-term and long-term management of JDM in resource poor settings could enable appropriate timely interventions for better treatment outcomes.

## Conclusion

The demographic characteristics of JDM patients from Cape Town (median disease onset age of 6.75 years and Female to male ration of 1.8:1) was similar to that reported from most regions of the world. It is probable that there was unnecessary delay in the diagnosis of some of the cases and in one instance, diagnosis was made retrospectively seven years after disease onset. Further, there was persistent clinically active disease in a large proportion of this cohort (60 %), putting them at risk of further disease complications. This could be a reflection of inadequate or less aggressive treatment. In concurrence with findings by other authors, this study found a relatively high rate of calcinosis among African patients with JDM. A prospective study to determine the factors associated with this heightened risk of calcinosis could be useful. The study adds to the available literature on JDM in Africa and demonstrates a need for a defined standard of care package to ensure adequate access to skilled multidisciplinary teams, essential diagnostic facilities and therapeutic resources for better treatment outcomes.

The study limitations included missing values and the relatively small number of cases identified. Further, we used an unvalidated modification of the PRINTO criteria for determination of disease activity which may raise questions on comparability of our results to findings from other studies. Establishment of a prospective cohort in future could be useful in providing better quality data for better outcome assessments in line with currently accepted international guidelines.

## Abbreviations

AST: Aspartate transaminase
CK: Creatine kinase
CMAS: Childhood myositis assessment scale
CRP: C-reactive protein
CT Scan: Computerised tomography scan
EMG: Electromyography
ESR: Erythrocyte sedimentation rate
IMACS: International myositis and clinical studies
IVIG: Intra-venous immune globulin
JDM: Juvenile dermatomyositis
LDH: Lactate dehydrogenase
MAA: Myositis associated antibodies
MDI: Myositis damage index
MMT: Manual muscle testing
MRI: Magnetic resonance imaging
MSA: Myositis specific antibodies
PRINTO: Paediatric rheumatology international trials organization
vWF: Von Willebrand factor
